# Non-coding RNAs derived from the foot-and-mouth disease virus genome trigger broad antiviral activity against coronaviruses

**DOI:** 10.3389/fimmu.2023.1166725

**Published:** 2023-03-29

**Authors:** Miguel Rodríguez-Pulido, Eva Calvo-Pinilla, Miryam Polo, Juan-Carlos Saiz, Raúl Fernández-González, Eva Pericuesta, Alfonso Gutiérrez-Adán, Francisco Sobrino, Miguel A. Martín-Acebes, Margarita Sáiz

**Affiliations:** ^1^ Centro de Biología Molecular Severo Ochoa, Consejo Superior de Investigaciones Científicas (CSIC)-Universidad Autónoma de Madrid (UAM), Madrid, Spain; ^2^ Department of Biotechnology, Instituto Nacional de Investigación y Tecnología Agraria y Alimentaria, Consejo Superior de Investigaciones Científicas (INIA-CSIC), Madrid, Spain; ^3^ Animal Reproduction Department, Instituto Nacional de Investigación y Tecnología Agraria y Alimentaria, Consejo Superior de Investigaciones Científicas (INIA-CSIC), Madrid, Spain

**Keywords:** non-coding RNA, foot-and-mouth disease virus (FMDV), SARS-CoV-2, COVID-19, type-I IFN, antiviral immunity, RNA-based therapy, coronaviruses

## Abstract

Severe acute respiratory syndrome coronavirus 2 (SARS-CoV-2) is the causative agent of a potentially severe respiratory disease, the coronavirus disease 2019 (COVID-19), an ongoing pandemic with limited therapeutic options. Here, we assessed the anti-coronavirus activity of synthetic RNAs mimicking specific domains in the non-coding regions of the foot-and-mouth disease virus (FMDV) genome (ncRNAs). These molecules are known to exert broad-spectrum antiviral activity in cell culture, mice and pigs effectively triggering the host innate immune response. The ncRNAs showed potent antiviral activity against SARS-CoV-2 after transfection in human intestinal Caco-2 and lung epithelium Calu-3 2B4 cells. When the *in vivo* efficacy of the FMDV ncRNAs was assessed in K18-hACE2 mice, administration of naked ncRNA before intranasal SARS-CoV-2 infection significantly decreased the viral load and the levels of pro-inflammatory cytokines in the lungs compared with untreated infected mice. The ncRNAs were also highly efficacious when assayed against common human HCoV-229E and porcine transmissible gastroenteritis virus (TGEV) in hepatocyte-derived Huh-7 and swine testis ST cells, respectively. These results are a proof of concept of the pan-coronavirus antiviral activity of the FMDV ncRNAs including human and animal divergent coronaviruses and potentially enhance our ability to fight future emerging variants.

## Introduction

The emergence in late 2019 of severe acute respiratory syndrome coronavirus 2 (SARS-CoV-2), a member of the genus *Betacoronavirus*, and its rapid global spread led the World Health Organization (WHO) to declare its associated disease, coronavirus disease 2019 (COVID-19) a pandemic in March 2020 (1). COVID-19 can cause severe disease and death with devastating health, social and economic impacts. At this time, the disease has resulted in over six million deaths having also unknown long-term consequences in people of all ages (https://covid19.who.int/) (2). While several COVID-19 vaccines are now available and have proven to be effective and life-saving, vaccine administration around the world remains insufficient and the duration of vaccine-induced immunity is still uncertain. Also, the constant emergence of new variants stands as a challenge for the vaccines which will likely require constant reformulation. This fact and the lack of effective antiviral therapies still threaten to overwhelm health care systems. Currently, only a few direct antiviral compounds have been approved by the US Food and Drug Administration (FDA) and the European Medicines Agency (EMA) for COVID-19 treatment including remdesivir, molnupiravir and ritonavir/nirmatrelvir (3–8). However, drug interactions and the emergence of antiviral-resistant strains remain a major concern. In this scenario, the identification of novel and effective antivirals with a different mode of action needs to be strengthened.

The ncRNAs are small and non-infectious synthetic RNA transcripts which mimic in sequence and structure three different domains in the non-coding regions (NCRs) flanking the genome of the foot-and-mouth disease virus (FMDV): the 5´-terminal S fragment (S), the internal ribosome entry site (IRES) and the 3´ non-coding region (3´NCR) (9, 10). These molecules are known to elicit a robust antiviral effect based on type I interferon (IFN) induction through both Toll-like and retinoic acid-inducible gene-I (RIG-I)-like receptors (TLR and RLR, respectively) signaling pathways (10, 11). The ncRNAs have been assayed against a variety of viral pathogens including zoonotic viruses causing severe disease in humans. Their sequence and structure triggered an antiviral state in mice able to prevent viral spread and disease after infection with FMDV (*Picornaviridae*) (12), West Nile virus (*Flaviviridae*) (13) and Rift Valley fever virus (*Phenuiviridae*) (14). We have also recently shown the antiviral activity of the ncRNAs against a complex DNA virus, African swine fever (ASFV, *Asfarviridae*) (15). The immunomodulatory role of these molecules has been assayed in co-administration with a conventional inactivated FMD vaccine. Direct inoculation of the IRES transcripts in pigs in combination with the vaccine had an enhancing effect on the specific B- and T-cell mediated immune responses elicited, also increasing the rate of protection against FMDV challenge (16).

In this study, we show the strong protective effect of the ncRNAs against SARS-CoV-2. Transfection of human intestinal and lung epithelium cells with the ncRNAs triggered the induction of type-I interferon (IFN) and IFN-stimulated genes (ISG) linking the activation of innate immune response with the measurable antiviral activity exerted. When the effect of the ncRNAs was assessed in a mouse model of COVID-19, delivery of naked IRES transcripts in K18-hACE2 transgenic mice before SARS-CoV-2 infection reduced viral load and inflammation in the lungs. The efficacy of the ncRNAs (S, IRES and 3´NCR) was also proved against infection by the common cold human coronavirus HCoV-229E and the swine coronavirus transmissible gastroenteritis virus (TGEV), both members of the genus *Alphacoronavirus*. Our results provide a proof of concept for the potential use of the FMDV ncRNAs in broad-spectrum strategies against both current circulating and future emerging coronaviruses.

## Materials and methods

### Viruses and cells

SARS-CoV-2 isolates MAD6, kindly provided by Luis Enjuanes (CNB, Madrid, Spain) and hCoV-19/Spain/SP-VHIR.02, D614G(S) from lineage B.1.610, kindly provided by Miguel Chillón (Universitat Pompeu i Fabra, Barcelona, Spain) were propagated in Vero E6 (African green monkey kidney epithelial cells, ATCC, CRL-1586). Cell supernatants were collected at 72 hpi, clarified, aliquoted and stored at -80C. The titers of the viral stocks were determined as 1 x 10^7^ PFU/ml for MAD6 and 1 x 10^6^ PFU/ml for hCoV-19/Spain/SP-VHIR.02, D614G(S) by standard plaque assay in Vero E6 cells. Human bronchial epithelial cells with increased expression of ACE2, Calu-3 2B4 (17) were kindly provided by Kent Tseng, UTMB, USA and Luis Enjuanes. Caco-2 (human colon epithelial cells, ATCC, HTB-37) and Calu-3 2B4 cells were infected with SARS-CoV-2 viral stock and passage 2 on each cell line was titered in Vero E6 (1.8 x 10^4^ PFU/ml and 8 x 10^6^ PFU/ml, respectively). The common cold coronavirus 229E expressing GFP (HCoV-229E-GFP) was kindly given by Volker Thiel, University of Bern, Switzerland and Antonio Alcamí, CBMSO, Spain. HCoV-229E-GFP was propagated in human liver Huh-7 cells (kindly given by Esteban Domingo, CBMSO, Spain). TGEV (kindly provided by Luis Enjuanes) was propagated in swine testis ST cells (ATCC, CRL-1746). Vesicular stomatitis virus (VSV) Indiana strain was propagated in human lung A549 cells (ATCC, CCL-185). Cells were cultured at 37°C with 5% CO_2_ in Dulbecco’s Modified Eagle Medium (DMEM, Gibco) supplemented with 10% fetal bovine serum (20% for Caco-2 and Calu-3 2B4 cells), 2mM L-glutamine and 1% penicillin/streptomycin (0.5% for Huh-7 and Caco-2 cells). For Huh-7, Calu-3 2B4 and Vero E6 culture DMEM was supplemented with 1% non-essential amino acids, and also with 25 µg/ml gentamycin for Huh-7 cells. For HCoV-229E-GFP propagation, Huh-7 cells were maintained at 33°C with 5% CO_2_.

### RNA synthesis, transfection and RT-PCR

RNA transcripts corresponding to the FMDV S, IRES or 3´NCR (404, 476 and 188 nt, respectively) were generated by *in vitro* transcription as previously described (10) with T3 (S and 3´NCR) or T7 (IRES) RNA polymerase (NEB), treated with RQ1 DNase, extracted with phenol-chloroform, precipitated with ethanol and finally resuspended in RNase-free water. The resultant 5´ppp-RNAs were quantified in a NanoDrop spectrophotometer ND-1000 (Thermo Scientific) and analyzed for size and integrity by electrophoresis. Prior to transfection, RNAs were denatured/renatured by heating at 92°C for 5 min and incubation at room temperature for 10 min in a concentrated 3-fold buffer to a final concentration of 10 mM Tris, pH 7.8, 1.5 mM MgCl2, 300 mM KCl (to keep the native RNA structure), and then chilled on ice. Transfection was performed using Lipofectamine 2000 (Invitrogen). Approximately 1 x 10^6^ Caco-2 or Calu-3 2B4 cells were transfected with 10 - 0.1 µg/ml 3´NCR, S or IRES transcripts. Transfection with *E. coli* MRE600 tRNA (Roche) or the dsRNA analog poly(I:C) (Invivogen) (5, 10 or 20 μg/ml) was used as negative or positive controls, respectively. For RT-PCR analysis, Caco-2 or Calu-3 2B4 cells were harvested at different times after transfection with 20 µg/ml 3´NCR. Then, total cytoplasmic RNA was extracted, quantified by spectrometry and treated for DNA removal with Turbo DNA-free kit (Ambion). Next, RNA aliquots (20 - 200 ng) were used for amplification of human IFN-β, Myxovirus resistance gene 1 (Mx1) or GAPDH mRNA. Primers used for IFN-β amplification (329 bp) were: 5´-GACATCCCTGAGGAGATTAAGCAGCTG-3´ and 5´-AGGCACAGTGACTGTCCTCCTTGG-3´. Primers for Mx1 amplification (299 bp) were: 5´-ACCAGAGTGGCTGTGGGCAA-3´ and 5´-ATCATGTAACCCTTCTTCAG-3´. PKR was amplified (1725 bp) with primers 5´-CGGCGTCTAGAATGGCTGGTGATCTTTCAGC-3´and GCGCGTCTAGACTAACATGTGTGTCGTTC-3´ (kindly provided by Iván Ventoso). GAPDH mRNA was amplified for normalization using previously described primers (11). Amplification products were analyzed by electrophoresis on agarose gels.

### Antiviral activity assays in Caco-2 and Calu-3 2B4 cells

The antiviral activity induced in Caco-2 or Calu-3 2B4 cells by transfection with the FMDV ncRNAs was determined as follows: approximately 10^6^ cells were transfected with the corresponding ncRNA (S, IRES or 3´NCR) at 20, 10, 1 or 0.1 μg/ml for 24 h at 37°C and then infected with SARS-CoV-2 at an MOI of 0.01, 0.1 or 0.5. Viral supernatants were collected at 96 hpi and then titered in Vero E6 cells by plaque assay after 4 days of infection. Viral titers were expressed as PFU/ml and compared with those in mock-transfected cells.

The paracrine antiviral activity induced by transfection with the ncRNAs was tested against VSV infection. For that, Caco-2 or Calu-3 2B4 cells were transfected with 20 µg/ml of 3´NCR transcripts for 24 h. Then, dilutions of transfection supernatants were used to treat human A549 cells (approximately 10^6^ cells) for 24 h prior to infection with VSV (100 PFU). In these assays, poly(I:C) and tRNA were transfected at the same concentration mentioned above (20 µg/ml), as positive and negative control, respectively. In some cases, supernatants were previously incubated for 1 h at 37°C with 1 μg/ml of human anti-IFN-α monoclonal antibody (clone MMHA-2, PBL Assay Science). The paracrine antiviral activity titers were expressed as the reciprocal of the highest dilution of supernatants required to reduce the number of VSV plaques by 50% in A549 cells.

### HCoV-299E antiviral assay in Huh-7 cells

Approximately 10^6^ Huh-7 cells were transfected with 20 μg/ml of each FMDV ncRNA (S, IRES or 3´NCR), poly(I:C) or tRNA and the supernatants were collected 24 h later for paracrine antiviral activity assay against human coronavirus-229E expressing Green Fluoresce Protein (HCoV-229E-GFP). For that, fresh Huh-7 cells in quadruplicate wells of 96-well plates were incubated for 24 h with dilutions of transfection supernatants and then infected with 10 tissue culture infectious dose 50 (TCDI_50_) of HCoV-229E-GFP in 100 μl per well. The viral titer of HCoV-229E-GFP in Huh-7 cells was 3 x 10^5^ TDCI_50_/ml. The plates were incubated for another 3 - 4 days when cytopathic effect (CPE) was observed in the non-treated wells (virus only). The number of GFP+ wells was then quantified under a Leica DM L3000 inverted fluorescent microscope and the antiviral activity titer was expressed as the reciprocal of the highest supernatant dilution reducing GFP expression (number of GFP+ wells).

### TGEV antiviral assay in ST cells

To test the antiviral activity induced in transfected cells approximately 10^6^ porcine ST cells were transfected with 20 µg/ml of S, IRES or 3´NCR transcripts, poly(I:C) or tRNA and 24h later infected with TGEV (50 - 100 PFU). CPE was monitored 48 h later by plaque assay and compared with that in mock-transfected cells. In some experiments, the antiviral effect of transfection with 20 µg/ml IRES transcripts for 0, 3, 6, 9 and 24 h was analyzed independently. The antiviral activity titer was expressed as the percentage of reduction in the number of TGEV plaques in RNA-transfected cells relative to mock-transfected cells.

The paracrine antiviral activity in supernatants from ST cells transfected with 20 µg/ml of S, IRES or 3´NCR transcripts, poly(I:C) or tRNA for 24 h was tested against TGEV infection. For that, dilutions of transfection supernatants were used to treat fresh ST cells for 24 h prior to infection with TGEV (50 -100 PFU). As above, the antiviral activity in supernatants of ST cells transfected with 20 µg/ml IRES transcripts for 0, 3, 6, 9 and 24 h was also analyzed by plaque assay 48 h after infection. The paracrine antiviral activity titers were expressed as the reciprocal of the highest dilution of supernatants required to reduce the number of TGEV plaques by 50% in ST cells.

### Immunoblot

Caco-2 or Calu-3 2B4 cells transfected with 20 µg/ml 3´NCR transcripts were washed twice in ice-cold PBS and lysed at different times after transfection in PBS containing 1% NP-40, 1mM DTT and 1X Complete protease inhibitor cocktail (Roche). Cell extracts (20 - 40 µg) were analyzed on 10% SDS-PAGE gels, transferred onto nitrocellulose membrane and probed with the following primary antibodies: anti-IRF3 mouse monoclonal antibody (SL-12, Santa Cruz Biotech), anti-Phospho-IRF3 (Ser396) rabbit monoclonal antibody (4D4G, Cell Signaling), anti-PKR rabbit polyclonal antibody (K-17, Santa Cruz Biotech), anti-ISG15 mouse monoclonal (F-9, Santa Cruz Biotech) anti-IL-6 mouse monoclonal (L-4, Santa Cruz Biotech), anti-MDA5 goat polyclonal (C-16, Santa Cruz Biotech) and anti-α tubulin mouse monoclonal (Clone B-5-1-2, Sigma-Aldrich) antibodies were used. Then, incubation with horseradish peroxidase-conjugated anti-rabbit, anti-goat (Invitrogen) or anti-mouse (Thermo Fisher Scientific) IgG allowed detection of membrane-bound proteins by chemiluminescent detection (NZY standard ECL, NZYTech).

### Antiviral efficacy assays in mice

A total of 21 six-week-old hemizygous K18-hACE2 transgenic female mice in two independent experiments were used. Animals were inoculated intraperitoneally (i.p.) with 200 µl containing 200 µg of ncRNAs diluted in PBS or PBS alone (vehicle) the day before infection (day -1). Prior to inoculation, IRES transcripts were heated at 92°C for 5 min, diluted in PBS and renatured at room temperature for 10 min. At day 0, animals were anesthetized under isoflurane and inoculated intranasally with 50 µL of DMEM containing 5 × 10^4^ PFU of SARS-CoV-2 isolate hCoV-19/Spain/SP-VHIR.02, D614G(S) (18). Mice were monitored daily for weight and clinical signs. All animals were anesthetized and humanly sacrificed at 3 dpi. Right mice lungs were harvested, homogenized in TriReagent (Thermo Fisher Scientific) using a TissueLyser II equipment and RNA was extracted from with RiboPure RNA Purification Kit (Thermo Fisher Scientific).

### RT-qPCR

The amount of SARS-CoV-2 genomic RNA was determined by one-step quantitative RT-PCR using the previously published primers CoV-F3, CoV-R3 and probe (19) and a QuantStudio5 Real-time PCR system (Applied Biosystems). Data are expressed as PFU equivalents/g of tissue by comparison with previously titrated samples. For cytokine expression analyses, the first strand cDNA was synthesized from total RNA extracted from mice lungs using Biotools High Retrotrasncriptase Starter Kit with Oligo dT (Biotools). The relative quantification of pro-inflammatory cytokines was performed by the 2^-ΔΔCt^ method using GAPDH as a housekeeping gene and the following PrimeTime Std qPCR Assays (Integrated DNA Technologies): Mm.PT.39a.1 for *GAPDH*, Mm.PT.58.10005566 for *IL-6*, Mm.PT.58.12575861 for *TNF-α*, Mm.PT.58.41616450 for *IL-1β*, Mm.PT.58.30682575 for *TIMP-1* and Mm.PT.58.43575827 for *Cxcl10*, and Mm.PT.58.10773148.g for *Cxcl11*. Data were expressed as fold change over the control (ncRNA/vehicle). The expression of hACE2 mRNA in the lung and trachea of transgenic mice was analyzed in RNA samples automatically extracted from mouse tissues using RNAeasy Mini Kit (Qiagen) and Quiacube equipment (20) as previously described (21).

### Statistical analysis

All experiments were performed in duplicates, triplicates or quadruplicates. Data are shown as mean ± S.D. Statistical analysis was performed using IBM SPSS Statistics v.28 and GraphPad Prism v.6. software. Comparisons were performed using two-tailed Student’s t-test.

## Results

### The FMDV ncRNAs are potent antivirals against SARS-CoV-2 in human cells

**Figure 1:**
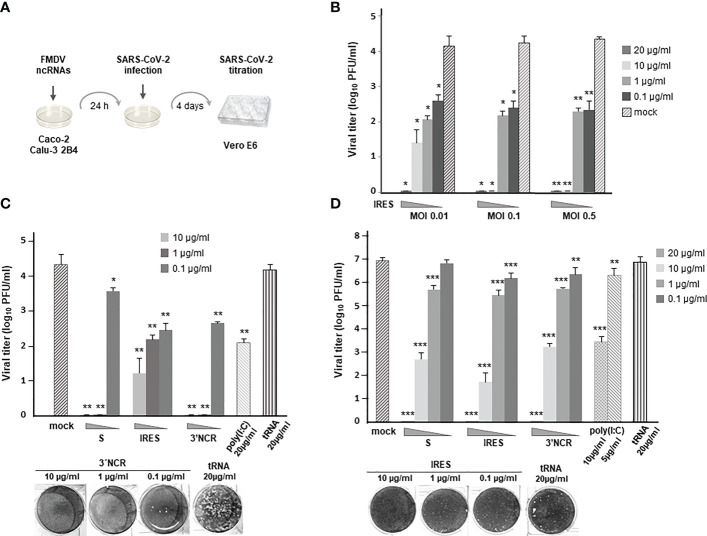
Antiviral activity induced by transfection of the FMDV ncRNAs in Caco-2 and Calu-3 2B4 cells. **(A)** Overview of the antiviral activity assay. **(B)** Caco-2 cells were transfected with IRES transcripts at the indicated concentration (20, 10, 1 or 0.1 μg/ml) for 24 h or mock-transfected. Then, cells were infected with SARS-CoV-2 at different MOI (0.01, 0.1 or 0.5). Supernatants were collected 4 days after infection and viral titers were determined in Vero E6 cells by plaque assay; Caco-2 **(C)** or Calu-3 2B4 **(D)** cells were transfected with S, IRES or 3´NCR transcripts, poly(I:C) or tRNA at the indicated concentration or mock-transfected and 24 h later infected with SARS-CoV-2 at an MOI of 0.1. Viral titers were determined as in **(B)**. VeroE6 cell monolayers illustrating some of the plotted data are shown. Data are mean ± SD of triplicates. Statistically significant differences between viral titers in RNA-transfected and the corresponding mock-transfected cells are indicated by asterisks (*p *<* 0.05, **p *<* 0.01, ***p *<* 0.001).

With the aim of testing the inhibitory effect of the ncRNAs on SARS-CoV-2 infection we first assayed the protecting effect of transfection with IRES transcripts in Caco-2 cells (**Figure 1A**). For that, cells were transfected with different amounts of RNA and 24 h later infected with SARS-CoV-2 at different multiplicity of infection (MOI). Four days after infection, supernatants from transfected and infected cells were collected and viral titers determined by plaque assay in Vero E6 cells (**Figure 1A**). The production of infectious SARS-CoV-2 progeny was found to be reduced in a dose-dependent manner in IRES-transfected cells (**Figure 1B**), showing a clear inhibitory effect, mostly blocking infection with 10 - 20µg/ml of RNA even at the highest MOI assayed (MOI of 0.5). Transfection with RNA doses as low as 1 - 0.1 µg/ml still induced a >100-fold reduction in viral titers at an MOI of 0.5 with very similar levels of protection against infection at an MOI of 0.1.

Next, the antiviral effect of all three ncRNAs (S, IRES and 3´NCR) transfected at 10, 1 or 0.1 µg/ml was assayed against SARS-CoV-2 (MOI of 0.1). Comparing the viral titers produced in Caco-2 cells that had been previously transfected with each ncRNA, the S and the 3´NCR transcripts conferred the highest inhibitory effect against SARS-CoV-2 infection at 10 and 1 µg/ml (**Figure 1C**). However, at the lowest RNA dose transfected (0.1 µg/ml), IRES and 3´NCR RNAs were about 10 times more inhibitory than S transcripts (**Figure 1C**). In these assays the dsRNA analog poly(I:C), known to inhibit infection of SARS-CoV-2 and other HCoVs in human cultured cells (22, 23), was included and its efficacy impairing SARS-CoV-2 infection was compared with that exerted by the different ncRNAs. As shown in **Figure 1C**, in cells transfected with 20 µg/ml of poly(I:C) the reduction of viral titers was remarkably lower (1 - 2 log) than that observed in Caco-2 cells transfected with each ncRNA. Transfection with 20 µg/ml of *E. coli* tRNA, as expected, did not affect viral titers.

To further evaluate the antiviral potential of the FMDV ncRNAs in a second biologically relevant cell culture system, we analyzed their effect against SARS-CoV-2 infection in human lung epithelium Calu-3 2B4 cells. As in Caco-2 cells, no infectious virus could be recovered from Calu-3 2B4 cells transfected with 20 µg/ml of each ncRNA, while 3 to 5-log reductions in viral titers were measured at 10 µg/ml, exerting the IRES transcripts the highest inhibitory effect (**Figure 1D**). Transfection with 1 µg/ml induced a reduction of 16 - 30 fold in viral growth, while 0.1 µg/ml had a milder effect, with significant reductions of 3.8- and 5.6-fold observed for the 3´NCR and IRES RNAs, respectively (**Figure 1D**). A 3000-fold reduction in SARS-CoV-2 titers was observed after transfection with 10 µg/ml of poly(I:C), while only a 4-fold reduction was achieved with a 5 µg/ml dose. As above, transfection with 20 µg/ml of *E. coli* tRNA did not affect viral titers. The IRES RNAs showed no cytotoxicity at 20 µg/ml in Calu-3 2B4 cells as determined by MTT assay (**Figure S1**). Altogether, these results show that transfection with the FMDV ncRNAs induce a robust antiviral activity against SARS-CoV-2 in human colon and lung cells.

**Figure 2:**
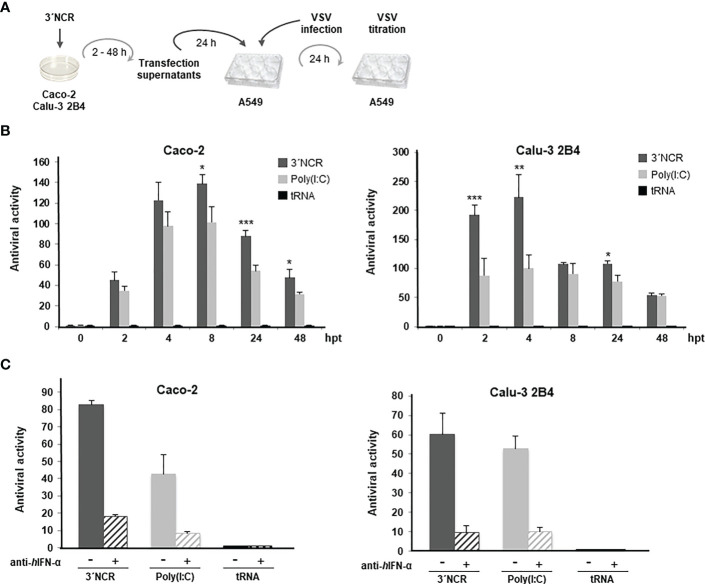
Paracrine antiviral activity induced by the 3´NCR transcripts in Caco-2 and Calu-3 2B4 cells. **(A)** Overview of the antiviral activity assay; **(B)** Caco-2 or Calu-3 2B4 cells were transfected with 20 μg/ml 3´NCR transcripts, poly(I:C) or tRNA and supernatants were collected at the indicated times after transfection. Then, A549 cells were incubated with dilutions of supernatants for 24 h before infection with VSV. The antiviral activity titers were expressed as the reciprocal of the highest dilution of supernatants reducing the number of VSV plaques by 50%. Data are mean ± SD of triplicates. Statistically significant differences between 3´NCR- and poly(I:C)-transfected cells are indicated by asterisks (*p *<* 0.05, **p *<* 0.01, ***p *<* 0.001). **(C)** The blockade of the antiviral activity in supernatants from Caco-2 or Calu-3 2B4 cells transfected with 3´NCR transcripts, poly(I:C) or tRNA for 24 h as above was assayed by incubation for 1 h with 1 μg/ml of human anti-IFN-α antibody. Data are mean ± SD of triplicates and titers are expressed as in **(B)**.

Next, the paracrine antiviral effect of transfection with the FMDV ncRNAs was measured by a standard IFN bioassay based on the inhibition of vesicular stomatitis virus (VSV) infection (highly affected by type-I IFN) (24) in human lung A549 cells (**Figure 2A**). Briefly, supernatants from Caco-2 or Calu-3 2B4 cells were collected at different times after transfection with 20 µg/ml of the ncRNAs and used to treat A549 cells that were 24 h later infected with VSV. In this case, only the 3´NCR transcripts were tested and compared with the effect of transfection with poly(I:C) or tRNA. As shown in **Figure 2B**, high levels of antiviral activity were measured after transfection in both cell lines with maximal values at 8 h post-transfection (hpt) in Caco-2 cells and 4 hpt in Calu-3 2B4 cells. Interestingly, antiviral activity titers over 200 were detected in Calu-3 2B4 cells as early as 2 hpt. Four hours later, titers were reduced to a half and maintained up to 24 hpt. Titers around 50 were still detected in supernatants of both cell lines 48 h after transfection with the 3´NCR (**Figure 2B**). Again, transfection with poly(I:C) induced lower levels of antiviral activity than the FMDV ncRNA, particularly at early times after transfection in Calu-3 2B4 cells, and no antiviral activity was detected in supernatants from tRNA-transfected cells. Though a residual stimulatory effect due to the presence of active RNA in the supernatants at early times after transfection cannot be ruled out, these results suggest that the 3´NCR transcripts triggered the rapid secretion of antiviral factors, present and active in the supernatants of transfected human cells. To assess the contribution of type-I IFN in the paracrine antiviral activity measured after ncRNA transfection, supernatants from Caco-2 or Calu-3 2B4 cells transfected with the 3´NCR RNA for 24 h were incubated with an anti-IFN-α antibody prior to the antiviral activity assay. As shown in **Figure 2C**, treatment with the antibody caused a remarkable reduction (about 80%) in the antiviral activity of the supernatants in both cell lines, supporting the relevant role of type-I IFN in the anti-SARS-CoV-2 activity elicited by the FMDV ncRNAs.

### Activation of innate immunity in human cells transfected with the FMDV ncRNAs

**Figure 3:**
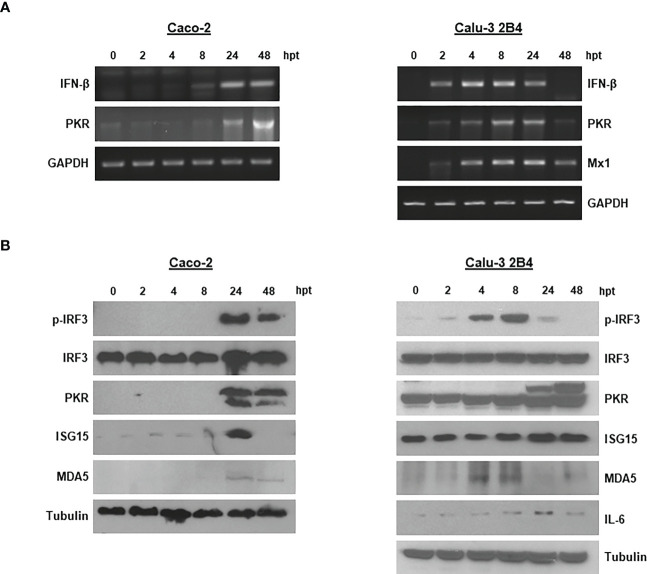
Innate immune response induced in Caco-2 and Calu-3 2B4 cells transfected with the 3´NCR. **(A)** Caco-2 or Calu-3 2B4 cells were lysed at different times after transfection with 20 μg/ml 3´NCR transcripts and induction of IFN-β, PKR and Mx1 mRNAs was analyzed by RT-PCR. GAPDH amplification was used for normalization. **(B)** The presence and levels of the indicated proteins were analyzed in lysates from Caco-2 or Calu-3 2B4 cells transfected as in **(A)** (up to 48 hpt) by immunoblot using specific antibodies.

Having proven the capacity of the ncRNAs to inhibit SARS-CoV-2 growth in Caco-2 and Calu-3 2B4 cells, and the involvement of IFN-α in the antiviral activity exerted, we examined the activation of relevant proteins in the IFN-I route and signaling pathway. For that, we first analyzed the transcriptional activation of IFN-β mRNA in 3´NCR-transfected cells. Induction of IFN-β mRNA was detected at 8 hpt in Caco-2 cells, while in Calu-3 2B4 it could be detected earlier, at 2 hpt, and up to 24 hpt (**Figure 3A**). The induction of PKR and Mx1, two IFN-stimulated genes (ISGs) of relevance in antiviral response, was also analyzed in cells transfected with the 3´NCR. As shown in **Figure 3A**, early induction of PKR mRNA could be detected in both Caco-2 and Calu-3 2B4 cells. Consistently, the induced synthesis and accumulation of PKR could be detected at 24 and 48 hpt in both cell lines by immunoblot (**Figure 3B**). However, Mx1 induction could only be detected in Calu-3 2B4 cells, unlike Caco-2 cells, from 2 hpt onwards and up to 48 hpt (**Figure 3A**), in agreement with previous data (25). The phosphorylated active form of IRF3 could be readily detected by immunoblot at 24 hpt in Caco-2 cells while it was detected as early as 4 hpt in Calu-3 2B4 cells (**Figure 3B**). A potent induction of ISG15 was also observed at 24 hpt in Caco-2, while a moderate induction of ISG15 and IL-6 could be detected at 24 - 48 hpt in Calu-3 2B4 cells transfected with the 3´NCR RNA (**Figure 3B**). IL-6 could not be detected in Caco-2 cells. Taking into account that MDA5 has been reported as the crucial viral sensor for triggering the antiviral response during SARS-CoV-2 infection (25, 26), we also analyzed the levels of MDA5 in Caco-2 and Calu-3 2B4 cells transfected with the 3´NCR transcripts. In fact, an early induction of MDA5 was detected by immunoblot at 4 hpt in Calu-3 2B4 cells while in Caco-2 cells induction of the RNA helicase was observed at later times after transfection (24 and 48 hpt, **Figure 3B**). Altogether, these results support the potent activation of the innate immune response induced in human cells susceptible to SARS-CoV-2 infection by transfection with the FMDV ncRNAs in agreement with the inhibitory effect against SARS-CoV-2 infection shown above.

### Infection of human and swine alphacoronaviruses is inhibited by the ncRNAs

**Figure 4:**
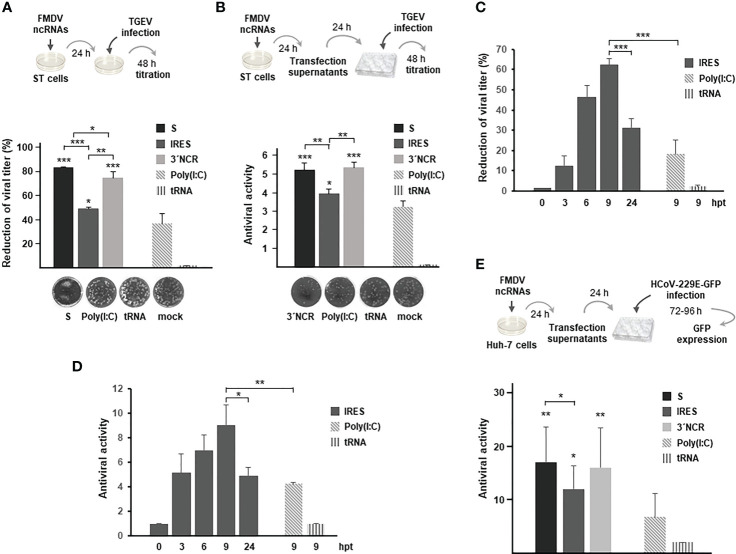
Inhibitory effect of the FMDV ncRNAs against TGEV and HCoV-229E. **(A)** Antiviral activity induced by transfection with 20 μg/ml S, IRES or 3´NCR transcripts, poly(I:C) or tRNA. Cells were infected 24 h after transfection with TGEV and incubated for 2 days for plaque assay. Antiviral activity was expressed as percentage reduction of viral titer. Data are mean ± SD of triplicate titrations from two independent experiments. ST cell monolayers transfected with the indicated RNA are shown. **(B)** Paracrine antiviral activity assay of supernatants from ST cells transfected for 24 h as in **(A)**. Dilutions of supernatants were used to treat fresh ST cell monolayers for 24 h before TGEV infection. CPE was monitored after 48 h by plaque assay. The antiviral activity titers were expressed as the reciprocal of the highest dilution of supernatants reducing the number of TGEV plaques by 50%. Data are mean ± SD of triplicates. ST cell monolayers showing the effect of treatment with a 1/5 dilution of the indicated supernatants are shown. In **(A, B)** statistically significant differences between ncRNAs- and poly(I:C)-transfected cells and those between S-, IRES- and 3´NCR-transfected cells are indicated by asterisks (*p *<* 0.05, **p *<* 0.01, ***p *<* 0.001); **(C)** ST cells were transfected with 20 μg/ml IRES for the indicated times and then infected with TGEV as above for plaque assay. The antiviral activity was expressed as percentage reduction of viral titer as in **(A)**; **(D)** paracrine antiviral activity titers in supernatants from ST cells transfected for the indicated times with 20 μg/ml IRES. Titers were expressed as in **(B)**. In **(C, D)**, ST cells were also transfected with 20 μg/ml poly(I:C) or tRNA for 9 h for comparison. Data are mean ± SD of triplicates. Statistically significant differences between IRES- and poly(I:C)-transfected cells at 9 hpt and those between IRES-transfected cells at 9 and 24 hpt are indicated by asterisks (*p *<* 0.05, ***p *<* 0.001); **(E)** paracrine antiviral activity in Huh-7 cells transfected with the ncRNAs against HCoV-229E. Huh-7 cells were transfected for 24 h with 20 μg/ml S, IRES or 3´NCR transcripts, poly(I:C) or tRNA. Then, supernatants were collected, diluted and used to treat fresh Huh-7 monolayers for 24 h before infection with HCoV-229E-GFP. The number of GFP+ wells was quantified and the antiviral activity titer was expressed as the reciprocal of the highest supernatant dilution reducing GFP expression after 3 - 4 days of infection. Data are mean ± SD of two independent experiments conducted in quadruplicate. Statistically significant differences between S- IRES- or 3´NCR-transfected and poly(I:C)-transfected cells are indicated by asterisks (*p *<* 0.05, **p *<* 0.01).

To test the broad-spectrum antiviral activity of the FMDV ncRNAs against coronaviruses, their protective effect was assayed against infection with TGEV and HCoV-229E alphacoronaviruses. TGEV causes a highly contagious, fatal gastroenteritis mainly in less than 2-week age piglets around the world (27). HCoV-229E causes mild to moderate upper-respiratory tract illnesses. When swine testis ST cells that had been previously transfected with the different ncRNAs were infected 24 h later with TGEV, a reduction in viral titers was observed in all cases, being also higher than the reduction induced by transfection with poly(I:C) (**Figure 4A**). In this case, the ncRNAs exhibiting the highest inhibitory effect were the S and the 3´NCR while the IRES transcripts seemed to be less efficient against TGEV infection. Similar results were obtained when the paracrine antiviral activity of the ncRNAs was assayed in ST cells against TGEV (**Figure 4B**). Again, the highest levels of inhibition were obtained when cells were treated with supernatants from cells transfected for 24 h with the S or 3´NCR transcripts, being the IRES RNA slightly less efficient in preventing TGEV infection under these experimental conditions. Next, we assayed the antiviral activity directly induced in transfected cells and the paracrine antiviral activity of the IRES RNA at different times after transfection in order to assess whether the IRES-exerted inhibition could be improved in terms of time of exposure to the RNA. As shown in **Figures 4C, D**, at 3 hpt in both assays antiviral activities were already detected and measured reaching the highest value at 9 hpt and decreasing at 24 hpt. These results suggest that the inhibitory effect exerted by the IRES transcripts, and likely the S and 3´NCR as well, could have been underestimated in the assays performed at 24 hpt (**Figures 4A, B**). Having seen that ncRNA delivery could inhibit infection by TGEV, we last tested their antiviral activity against HCoV-229E, a different alphacoronavirus causing influenza-like illness in humans. For that, a paracrine antiviral activity assay was performed in human liver Huh-7 cells. Treatment of Huh-7 cells with supernatants from ncRNA-transfected cells had a protective effect against HCoV-229E infection, in all cases significantly higher than that observed for poly(I:C) (**Figure 4E**). In this case, the inhibitory effect of the S and 3´NCR RNAs was very similar while slightly higher than that exerted by the IRES transcripts. As a whole, these results support the broad range of protection against infection by coronaviruses exerted by the FMDV ncRNAs.

### Delivery of naked ncRNA reduces viral load and inflammation in K18-hACE2 mice

The protective effect of the ncRNAs against SARS-CoV-2 infection was assessed in a COVID-19 mouse model. For that, transgenic mice expressing the human angiotensin-converting enzyme 2 (hACE2) under the control of the human cytokeratin 18 (K18-hACE2) were generated (**Figure S2**). K18-hACE2 mice support SARS-CoV-2 infection showing severe disease and pathogenesis in the lung (28–30).

**Figure 5:**
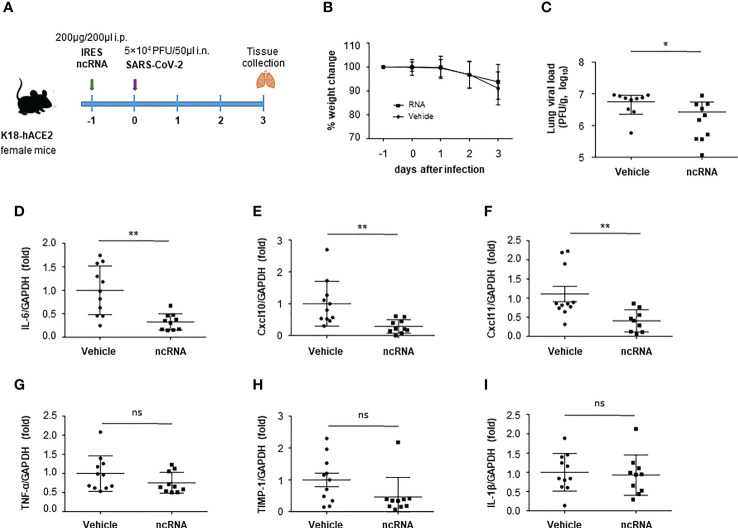
Effect of IRES treatment in a mouse model of COVID-19. **(A)** Experimental timeline. Six-week-old female K18-hACE2 transgenic mice were inoculated i.p. with 200 μg IRES transcripts or PBS (vehicle). One day later mice were infected *via* the intranasal route with 5 x 10^4^ PFU of SARS-CoV-2. Body weight (pooled) was evaluated daily (mean ± SD) **(B)**. At 3 dpi, mice were euthanized and the lung viral load **(C)**, interleukin-6 (IL-6) **(D)**, C-X-C motif chemokine ligand 10 (CXCL10) **(E)**, C-X-C motif chemokine ligand 11 (CXCL11) **(F)**, TNF-α **(G)**, TIMP-1 **(H)** and IL-1β **(I)** levels in lung were determined by RT-qPCR. Plotted data are means ± SD of two independent experiments. Statistically significant differences between RNA-inoculated and vehicle groups are indicated by asterisks (*p < 0.05, **p < 0.01); ns, not significant.

Groups of K18-hACE2 mice were inoculated i.p. with a single dose of 200 µg IRES transcripts diluted in PBS or vehicle (PBS) 24 h before intranasal inoculation with 5 x 10^4^ PFU of SARS-CoV-2 (hCoV-19/Spain/SP-VHIR.02, D614G(S) (**Figure 5A**). On the third day after infection, mice were euthanized, lung tissue was collected and lung viral load and cytokine levels were evaluated. At 3 days post-infection (dpi) the viral load is expected to reach the highest levels in the lungs of K18-hACE2 mice infected with SARS-CoV-2 (29). The treatment with IRES RNA at this dose and up to day 3 after infection did not induce early weight loss or any other signs of toxicity whereas a slight reduction in weight loss was observed in the vehicle-inoculated group suggesting a protective effect against SARS-CoV-2 infection in IRES-inoculated mice (**Figure 5B**). To study the effect of ncRNA treatment in lungs, the most important target tissue for COVID-19 pathology, we evaluated the abundance of SARS-CoV-2 in mouse lung through RT-qPCR at 3 dpi. The average viral load in the IRES-treated group was significantly lower compared with the vehicle-treated mice (**Figure 5C**). We then investigated whether the decrease in viral load would have positive effects on lung inflammation (**Figure 5D–I**). The expression of transcripts encoding the pro-inflammatory cytokine interleukin-6 (IL-6) (**Figure 5D**) and the C-X-C motif chemokine ligand 10 (CXCL10) (**Figure 5E**) and 11 (CXCL11) (**Figure 5F**) was markedly diminished in RNA-treated mice, and a trend to reduction, though not reaching statistically significant values, was also observed for TNF-α (**Figure 5G**), Timp1 (**Figure 5H**) and IL1-β (**Figure 5I**). Together, our results show that prophylactic i.p. administration of naked IRES RNA can reduce SARS-CoV-2 replication and the production of inflammatory cytokines and chemokines in the lungs of K18-hACE2 mice.

## Discussion

The ongoing pandemic caused by SARS-CoV-2 has generated the need for efficient therapeutics for COVID-19 patients. The rapid evolution of the virus and the subsequent emergence of new variants have hampered the protective immunity elicited by vaccination or natural infection. To address an unmet medical need for the treatment of current human coronavirus infections and to maximize pandemic preparedness, broad-spectrum antiviral therapies that are effective against current and future emerging coronaviruses are needed. Antiviral treatments can exert their maximal effect at the early stages of infection reducing the symptoms and severity of the disease while their use as prophylactic drugs might fully prevent them. In this study, we evaluated the antiviral effect of the FMDV ncRNAs, synthetic non-coding RNA molecules derived from the FMDV genome, against infection by SARS-CoV-2 and other human and animal coronaviruses. Transfection of human colon and lung epithelial cells with any of the FMDV ncRNAs -3´NCR, S and IRES- prevented SARS-CoV-2 infection in a dose-dependent manner, fully blocking infection at 20 μg/ml (312 nM, 123 nM and 145 nM for 3´NCR, S and IRES transcripts, respectively) and with a milder, but still significant reduction effect in viral titers at 0.1 µg/ml. Though these assays did not include other SARS-CoV-2 variants of concern for which the protection levels might be slightly different, the broad-spectrum anti coronavirus capacity of these RNA molecules was tested against common cold HCoV-229E and swine TGEV alphacoronaviruses. On the other hand, any new information on their inhibitory effect against the currently circulating SARS-CoV-2 variants would further support their potential use as pan-coronavirus antiviral drugs. In these assays the IFN stimulatory activity of the ncRNAs, all carrying a 5´-triphosphate, was compared with that of the dsRNA analog poly(I:C) and *E. coli* tRNA used as positive and negative controls, respectively. It should be noticed that the 5´- triphosphate contributes to but is not strictly essential for the stimulatory activity of the FMDV ncRNAs, known by previous work to rely mainly on structural features in the RNA domains (10). However, the IFN-inducing capacity is not a conserved attribute among equivalent viral RNA motifs despite of their extensive secondary structure (presence of unmodified dsRNA) and 5´-triphosphate generated during *in vitro* transcription.

Sensing of viral RNA during SARS-CoV-2 infection triggers innate antiviral defense in the host cells that is efficiently counteracted through a variety of molecular mechanisms developed by the virus (31–34). Delivery of the FMDV ncRNAs into human colon and more importantly, lung epithelial cells triggered the transcription of IFN-β and ISG mRNAs inducing an antiviral state in transfected cells that could be detected and measured by immunoblot and paracrine antiviral activity assays. Remarkably, the expression of MDA5, an innate immune sensor involved in antiviral response against SARS-CoV-2 infection (25, 26), was clearly upregulated in ncRNA-transfected cells.

We demonstrated the *in vivo* efficacy of prophylactic ncRNA administration against SARS-CoV-2 using the K18-hACE2 mouse model (30). Inoculation of naked IRES transcripts 24 h before SARS-CoV-2 infection mitigated body weight loss, reduced viral titers and modulated the inflammatory response in lungs of K18-hACE2 mice. Though only the prophylactic use of the FMDV ncRNAs was assayed in this study, previous work showed increased protection in ncRNA-inoculated mice at 1 or 5 days after infection with WNV or RVFV (13, 14) suggesting their potential efficacy in a therapeutic setting, while requiring further *in vivo* research.

An early and robust IFN-I response to SARS-CoV-2 infection is essential for rapid control of viral replication (35–38). Consistently, therapeutic treatment with recombinant IFN has been shown beneficial when applied soon after the infection, while late treatment can be detrimental due to increased inflammation (39). Treatment with recombinant pegylated-IFN-α/β/λ improved the recovery of COVID-19 patients in clinical trials (40–42). Endogenous stimulation of the innate immune system prior to or after infection with SARS-CoV-2 is also being explored. Intranasal administration of poly(I:C) showed antiviral activity comparable with IFNα A/D in hamsters (43) and induced upregulation of innate immune response in lungs and increased survival in mice when administered immediately after SARS-CoV-2 challenge (44). Direct comparison of the *in vivo* anti-coronavirus activity of poly(I:C) and the FMDV ncRNAs will be of interest for future work. Several agonists of viral immune sensors RIG-I and STING have yielded better results than recombinant IFN in K18-hACE2 mice likely due to reduced production of anti-type I IFN autoantibodies and broader stimulation of antiviral effectors (45–48). Prophylactic treatment with a 14-bp RNA duplex at 16 or 2 h before infection protected mice from clinical disease after SARS-CoV-2 infection (45). Similarly, administration of a synthetic 5´-triphosphate dsRNA RIG-I ligand 1 day before SARS-CoV-2 infection increased the survival rate and reduced viral burden after challenge (46). In both cases, the RNAs were injected intravenously and complexed with a polyethylenimine-based transfection reagent. Here, we show that delivery of naked IRES RNA i.p. one day prior to infection with a lethal dose of SARS-CoV-2 significantly reduced viral titers and pro-inflammatory cytokines in the lungs of K18-hACE2 mice. Considering that the mouse model assayed exhibits 100% lethality, while fatality rate in humans remains around 1%, the protective effect of the ncRNAs in humans against SARS-CoV-2 may be underestimated. The moderate but significant reduction of viremia and inflammation in lungs observed might be widely beneficial for COVID-19 patients. Therefore, these results provide a proof of concept of the *in vivo* efficacy of the FMDV ncRNAs for the treatment of coronavirus diseases. However, additional data supporting this evidence will be required before their prophylactic or therapeutic use, specially aimed to address the protection window of the administration at different times before and after infection with different coronaviruses. The detailed characterization of the immune response including the immune cell profiling in lungs as well as the monitorization of cytokines in serum of ncRNA-treated mice at different times before and after infection will be addressed in future experiments. The combined use of high-throughput OMICS techniques, like RNA-seq and proteomics, in ncRNA-treated human cells would be of much interest and help understand the induced changes in gene expression profiling and their interference with the disease mechanisms caused by SARS-CoV-2. Unlike the engineered RIG-I or STING agonists above, the FMDV ncRNAs mimic specific viral regions acting as pathogen-associated molecular patterns recognized by multiple RNA sensing molecules (RIG-I, MDA5 and endosomal TLRs) being effective immunomodulators *in vivo* against a variety of human, animal and zoonotic viruses (12–14). Unlike most antivirals currently used against COVID-19 displaying high target specificity, the FMDV ncRNAs would act in a broad-based manner eliciting the antiviral response to prevent infection or hinder viral progression in a therapeutic setting. Their mode of action allows their combination with other specific drugs that might result in synergic formulations against SARS-CoV-2. Also, it may be worth exploring their use, alone or combined with other antivirals for viral clearance in long-COVID patients with SARS-CoV-2 persistence. Though future work will be required to compare their efficacy with other antiviral drugs, address the optimal dosing time and administration schedule to avoid immunopathology, as well as the appropriate dose and means of delivery for their therapeutic use in humans, our study provides solid evidence of the versatile efficacy of the FMDV ncRNAs against coronaviruses.

Altogether, our results support the use of the FMDV ncRNAs as broad-spectrum antivirals against new emerging viruses and prove their intrinsic efficacy *in vivo* as naked RNA in a lethal mouse model of COVID-19, paving the way for RNA-based combined therapeutic strategies that could potentially prevent infection by SARS-CoV-2 and newly emerging viruses.

## Data availability statement

The raw data supporting the conclusions of this article will be made available by the authors, without undue reservation.

## Ethics statement

The animal study was reviewed and approved by Ethical Committee of Animal Experimentation of INIA and by the Division of Animal Protection of the Comunidad de Madrid (PROEX 115.5-21).

## Author contributions

Conceptualization, MS. Methodology, MS, MR-P, AG-A and MM-A. Validation and Formal Analysis, MR-P and MM-A. Investigation, MR-P, MP, EC-P, RF-G, EP and MM-A. Resources, MS, FS, MM-A, AG-A and J-CS. Writing – original draft, MS, MR-P and MM-A. Writing-review & editing, MS, MR-P, EC-P, MP, FS, MM-A, AG-A, J-CS, RF-G and EP. Visualization, MS, MR-P and MM-A. Supervision, MS, MM-A and FS. Project administration, MS and MM-A. Funding acquisition, MS, FS. and MM-A. All authors contributed to the article and approved the submitted version.
