# Supplementary material for: Non-coding RNAs derived from the foot-and-mouth disease virus genome trigger broad antiviral activity against coronaviruses
Source: Front Immunol. 2023 Mar 29;14:1166725. doi: 10.3389/fimmu.2023.1166725 (PMC10090856; doi:10.3389/fimmu.2023.1166725)
Supplement: Supplementary file 1 [file DataSheet_1.pdf]

Supplementary Material

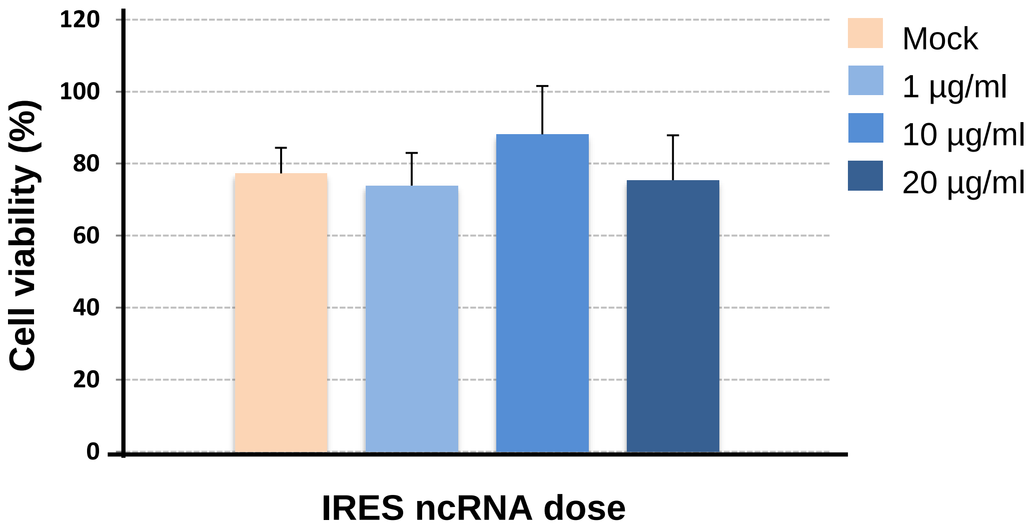

Figure S1. Cytotoxicity of IRES ncRNA in Calu-3 2B4 cells. Cell viability was analyzed by [4,5-dimethyl-2-thiazolyl]-2,5-diphenyl-2-H-tetrazolium bromide (MTT) assay in IRES-transfected cells. Calu-3 2B4 cells were seeded on 96-well plates and incubated for 24 h before transfection with 1, 10 or 20 µg/ml of RNA or mock-transfection using Lipofectamine 2000 (Invitrogen) as described in the Materials and Methods section. 24 h after transfection, 90 µl of the supernatant was removed and replaced with 90 µl of fresh medium containing 10 µl of MTT solution (7.5 mg/ml) diluted in PBS. Cells were incubated at 37ºC for 2 h in the dark and lysed with 100ul of lysis buffer (20% SDS, 0.45N acetic acid and 0.025 N HCl in N, N-dimethylformamide) for 15 min at room temperature. The absorbance was recorded at 550 nm using a microplate reader (Bio-Rad). Data are mean ± standard deviation of six independent transfections.

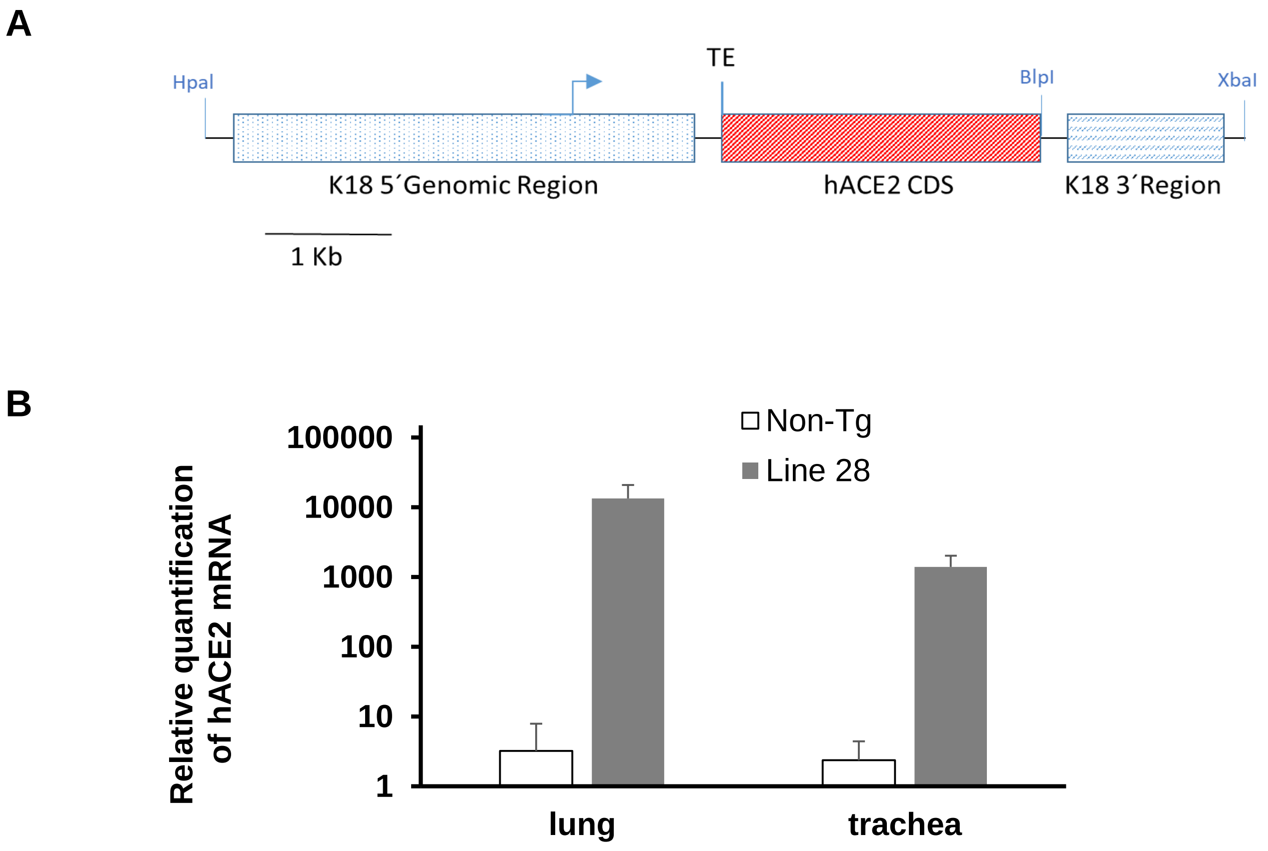

Figure S2. Generation and characterization of K18-h*ACE2* mice. A) Schematic view of the expression cassette of plasmid pK18-*hACE2* as described.[1] B) Relative quantification of *hACE2* expression in K18-hACE2 transgenic mice from Line 28 and control non-transgenic mice (Non-Tg). Transgene expression was calculated relative to GAPDH in Line 28 and Non-Tg by real-time RT-PCR in six-week old female mice (n=5).

Supplementary Methods

Generation and characterization of transgenic mice. Humanized transgenic mice expressing the human angiotensin-converting enzyme 2 (hACE2) under the control of the human cytokeratin 18 (K18-hACE2) were generated as previously described[2] using plasmid pK18-*hACE2*[1] that expresses the human angiotensin-converting enzyme 2 (hACE2) under the control of the human cytokeratin 18. pK18-*hACE2* was a gift from Paul McCray (Addgene plasmid # 149449; http://n2t.net/addgene:149449 ; RRID:Addgene_149449). B6CBAF1 mice (C57BL/6JOlaHsd x CBA/CaOlaHsd, Envigo, Netherlands) were used in this study to produce transgenic mice. All mice used in this experiment were housed under controlled temperature conditions of 23 °C with a 14:10 h light:dark cycle and free access to water and food. The transgene construct was excised from the vector by digesting with restriction endonucleases *Hpa*I and *Xba*I. A DNA fragment of 6.8 kb was obtained, purified using the QIAEX II Gel Extraction Kit (Quiagen Inc., Hilden, Germany) and re-suspended in 10 mM Tris, pH 7.4, with 0.1mM ethylene diamine tetraacetic acid (EDTA; TE) at a final concentration of 2 to 6 ng/mL. Finally, the transgene solution was microinjected into pronuclear-stage embryos collected from superovulated B6CBAF1 females.[3] DNA was extracted from tail biopsies of the resulting offspring and the presence of the transgene was identified by PCR amplification using specific primers for the transgenic construct (forward primer ACCTGGCTGAAAGACCAGAACAAG and reverse primer AATTAGCCACTCGCACATCC) to identify founders. For this study, the transgenic line 28, which exhibited the highest expression of hACE2 in the lungs was selected. Heterozygotes were used in all the experiments. The expression of hACE2 mRNA in lung and trachea of transgenic mice was analyzed by real-time PCR in RNA samples automatically extracted from mouse tissues using RNAeasy Mini Kit (Qiagen) and a Quiacube equipment as previously described.[1]

Supplementary References

1. McCray, P.B., Jr., Pewe, L., Wohlford-Lenane, C., Hickey, M., Manzel, L., Shi, L., Netland, J., Jia, H.P., Halabi, C., Sigmund, C.D., Meyerholz, D.K., et al. (2007). Lethal infection of K18-hACE2 mice infected with severe acute respiratory syndrome coronavirus. J Virol *81*, 813-821. 10.1128/JVI.02012-06.
2. Gutierrez-Adan, A., and Pintado, B. (2000). Effect of flanking matrix attachment regions on the expression of microinjected transgenes during preimplantation development of mouse embryos. Transgenic Res *9*, 81-89. 10.1023/a:1008926022370.
3. Gutierrez, A., Meade, H.M., Ditullio, P., Pollock, D., Harvey, M., Jimenez-Flores, R., Anderson, G.B., Murray, J.D., and Medrano, J.F. (1996). Expression of a bovine kappa-CN cDNA in the mammary gland of transgenic mice utilizing a genomic milk protein gene as an expression cassette. Transgenic Res *5*, 271-279. 10.1007/BF01972881.
